# The Association between Ground Floor Features and Public Open Space Face-To-Face Interactions: Evidence from Nantou Village, Shenzhen

**DOI:** 10.3390/ijerph16244934

**Published:** 2019-12-05

**Authors:** Mirna Zordan, Gianni Talamini, Caterina Villani

**Affiliations:** Department of Architecture and Civil Engineering, City University of Hong Kong, Hong Kong, Chinacvillani2-c@my.cityu.edu.hk (C.V.)

**Keywords:** street façade, face-to-face interaction, healthy city, correlational analysis, Kernel density maps

## Abstract

With face-to-face interaction proving beneficial for mental health, there is still a paucity of research on the correlation between ground floor features (GFFs), defined here as the features of the ground floor of buildings overlooking a street, and public open space face-to-face interaction density (POSFTFID), defined as the density of human face-to-face interactions in the public open space (POS) adjacent to each building. Is there a correlation between GFFs and POSFTFID? This study aims to answer this question gaining empirical evidence from a Chinese village in the city (ViC). Behavioural mapping and statistical analysis were employed and the following GFFs were tested: Ground floor area, indoor visible space, presence of stairs, POS adjacent area, and land use. Results show an association between POSFTFID and: (1) The area of the POS adjacent to each building, (2) the degree of visibility (ratio of indoor visible space to total internal space) of the ground floor, (3) the presence of stairs. Moreover, food appears to be an important attribute fostering social interaction. Results can provide insights on future implications in urban design strategies and planning policies aiming at enhancing mental health conditions in contemporary cities.

## 1. Introduction

Since the definition of health by the World Health Organization (WHO) in 1948—“a state of complete physical, mental and social well-being and not merely the absence of disease or infirmity”—a growing attention has been dedicated to the relationship between the urban environment and its impact on people’s behaviour and well-being. A vast quantity of research has already been conducted to investigate the effect of BE on physical health, focusing on the importance of physical activity for people’s well-being especially in relation to the prevention or the recovering from increasingly common diseases such as obesity [1,2,3,4,5,6,7,8,9], or cardiovascular diseases [3], among all populations (kids, teenagers, adults, and older adults).

Yet, the relationship between BE and mental health has been the focus of relatively limited research. Existing studies analysed the impact of various kinds of environments on mental health, particularly focusing on residential settings where researchers analysed housing typologies [10] and high rise buildings [11] in association with depression symptoms. Moreover, regarding outdoor environments, POS spatial attributes have been explored in association with mental health [12], green spaces have been investigated in association with cognitive functions [13], green and blue spaces have been studied in relation to mental benefits [14]. Another field of research analysed residential environmental characteristics and neighbourhood-associated factors [15]. Despite the growing body of knowledge on this topic, these fields of investigation present a wide potential for research exploration.

In the last few decades, relevant studies focused on social interactions and their impact on people’s mental health and well-being. Particularly, in the contemporary urban environments, where new technologies and media are expanding affecting human life and social communication, evidence has been gained to prove the prevalence of the positive impact of offline social interactions compared to computer media communication, or online interactions. These studies have shown the association between symptoms of depression with time spent online [16]. Caplan [17] confirms this theory, relating psychosocial well-being to social interactions rather than online communication. The benefits of social interactions are also relevant in absolute terms. It has also been found that social interactions have a significant impact on cognitive functions, stimulating brain activity [18]. Recent studies demonstrate the benefits of social interactions on public health, especially regarding mental health care [12]. One recent study investigated psychological well-being indicators in individuals engaging in social interactions compared with individuals not engaging in social interactions [19]. The results demonstrated that individuals experiencing social interactions have higher levels of psychological well-being. Moreover, social interactions play a fundamental role in the definition of social life and social capital [20], both of which are fundamental to people’s well-being. Eventually, social interactions can even support the prevention of premature mortality [21].

A critical component of social interaction is non-verbal communication [22]. In this study, face-to-face interaction is intended as both verbal and non-verbal communication. The area of study concerning face-to-face interaction still lack a formal definition [22,23,24]. Interactionism had been discussed in various fields of studies, among others, philosophy, sociology, psychology, and anthropology. Scholars such as Simmel, Blumer, Mead, and Goffman significantly contributed to conceptualizing this topic. The term “face-to-face interaction” comprehends social interactions occurring during both stationary [25,26] and necessary activities involving essential verbal communication [25]. Face-to-face communication plays an essential role in making individuals, in a face-to-face proximity distance, potential interactors [27,28]. In non-verbal communication, each interactor is a giver and/or a receiver [24]. The condition of face-to-face proximity distance fulfil an essential role in favouring non-verbal communication, and it enhances the chances for social interaction to occur.

Face-to-face interaction can happen in any type of space: Public, semi-public, and private [22]. Considering the importance in this paper of individuals as potential interactors, the chosen settings are urban streets devoted to pedestrian use. Many researchers have already analysed the role of the street as a source of public space that enhance urban dynamics and urban life. In urban studies, the debate on streets role and function is on-stream since Modernism, when Le Corbusier declared “the death of the street”, a principle that was rebutted by thinkers such as Kevin Lynch [29], Jane Jacobs [30], and Jan Gehl [26] that celebrated the importance of streets as fundamental players in enhancing human connectivity.

## 2. Literature Review

Urban streets have been the focus of relevant studies that attempt to relate their spatial characteristics with different aspects of human behaviour. The research mainly focused on the ability to gather and create more liveable space and higher walking behaviour. Studies on the correlation between streets’ spatial attributes and POSFTFID produced more mixed (and fewer) results. Although specific research on the design features correlated with social interactions is limited, notable research on the design characteristics that can influence pedestrians’ activity patterns or behaviour has been undertaken for the ground-level of urban streets [31,32,33,34]. The ground-level space between buildings’ GFFs and the linked public space has been systematically observed to be the setting for pedestrian activity and interaction since the 1980s [35]. Notable environment-behaviour studies found that in case of a direct connection between the outdoor ground-level public space and the indoor buildings’ façade, indoor activities tend to extend and take place in front of the building [36]. This condition was defined as active edges when “the frontage of a building at ground floor level with frequent doors and windows, details and articulation to the facade and visible internal uses” [37]. Similar conditions were referred to as “soft edges” [36], “transitional edges”, “public/private interfaces” [33,38], “interactive edges” [39], “in-between spaces” [40,41,42], and “staying zones” [34]. Relevant studies investigated several environmental and specific design features that have an impact on pedestrian social behaviour or activity at the ground level of streets. Although it is not possible to draw a clear distinction between environmental features and effects on pedestrian behaviour due to the interaction among features, the remainder of this part attempts to be categorize and summarize features ed effects in Table 1.

### 2.1. Architectural Articulation

The architectural articulation includes façade articulation, rhythm and scale, and the observed impacts are summarized in Table 1. Relevant studies associate pedestrians’ preference for moderate façade articulation (or complexity), expressed by the “unity and variety” of the facade [43]. The architectural features of the façade: texture, size, colour, façade components and shape, and the pedestrian or car setbacks [33,44,45] are observed to enhance the visual quality of urban settings [43]. In addition, when the dimensions and subspaces of the architectural elements match human proportions, the façade articulation can contribute to create a human scale environment. This is defined as “a fine scale characterized by the human body and its surroundings, … a scale that is directly visible, touchable, and appreciable in a person’s daily life” [46]. Similarly, the rhythm—presence of vertical and horizontal lines—and scale—the presence of narrow units—of the architectural façade can influence pedestrian behaviour. The first can contribute to the attraction of pedestrians’ attention to ground level and to lowering the walking speed, the latter can increase pedestrian activities through the provision of niches in the architectural front [45,47].

### 2.2. Permeability

The ground floor is defined as a spatial element characterized by different degrees of physical permeability and transparency [33]. The physical permeability of the ground floor consists of the degree of physical access to a space behind (indoor or private) the façade [33]. This is measured through the degree of street-front access at the block-segment, in the number of ground-level entrances and in the proportion of active uses (e.g., commercial) [48]. The transparency is the degree to which pedestrians can see human activity beyond the architectural front, standing from the street [33,45]. The variables referred to as transparency are mostly related to bi-dimensional attributes such as glass surfaces or presence of doors and windows in the first floor facing the street [45], or to the fragmentation and articulation of the façade, and edge zones [49]. In addition, Dovey and Wood [33] distinguish the degree of GFFs transparency in impermeable, opaque, and transparent. Both physical permeability and transparency contribute to creating or sustaining pedestrian activity, through a physical or visual exchange between indoor/outdoor inside/outside [49]. The higher degree of GFFs’ physical permeability can attract pedestrians’ attention to ground floor space [35]. Similarly, higher transparency together with more articulate façade can correspond to clustering of pedestrian activities [32,38].

### 2.3. Personalization

Personalization is the degree to which a street appears to be modified and claimed by residents or store owners [44]. This is observed in the presence of decorative elements or signs, the addition of (movable or fixed) seating and shading, the occurrence of vegetation or landscaped areas, and in the modification of buildings’ colours [44,48,50]. Personalization can be expressed in small modifications of ground floor space, such as the addition of makeshift seating space or extended vending stall [50]. This is observed to contribute to the sense of security to users, through providing opportunities to have “eyes on the streets” [30]. In addition, Mehta [48] suggests that personalization of ground-level street space might promote pedestrian activities in the premises of the modified space.

### 2.4. Enclosure

The enclosure is the degree to which public space is visually defined by buildings, walls, trees [34,39,45,48,49,51], and it is commonly described by the height to width ratio examining the proportion between the width of the sidewalks and the height of the building. The number of street furniture and vegetation, and the proportion of sky ahead of the pedestrians are also a commonly observed measurement of the sense of enclosure [45,52]. The sense of enclosure is related to pedestrians’ preference for enclosed spaces specifically at the ground level and this contributes to attracting pedestrians to the sidewalk [36,45,47].

### 2.5. Land Use

Several studies observed that the diversity in the ground-level land use, specifically the presence of commercial use can help initiate pedestrian activity (though providing services) or support the existing activity levels [36,53]. In predominantly residential neighbourhoods the presence of small-scale local and independent commercial businesses on the streets can positively influence the sense of security to users, providing a place where costumers are “watching over the streets and public spaces” [30,54]. The outdoor commercial seating space can be a meeting place thus stimulating the public social life [54].

### 2.6. Amenities Provision

The provision of amenities in the street level space consists in the availability of seating space, canopy sheltered, and shaded areas that can provide and increase the sense of pedestrian comfort, especially during weather seasonal changes. Whyte’s [31] observational research of public space use as a function of physical variables led to the main finding that sitting space is a highly significant variable in supporting pedestrians activity. The provision of amenities is observable in the number of public or commercial seating space at the block-segment level, and in the percentage of shaded and sheltered space (e.g., from trees and canopies) at the block-segment level [32,55]. Seating and shaded space is observed to catalyse pedestrian activity even when this space incidental and not purposely designed [50,56]. In addition to the contribution to pedestrians’ comfort, the promotion of pedestrian activities on the streets, the provision of amenities can increase the sense of human scale at the street level [38].

### 2.7. Limitations of Previous Studies

From the existing literature, some limitations can be found. In all previous studies, none is considering the allowance of spatial and visual interpenetration of open and closed spaces, or in other terms, the degree of visibility through it. As we mentioned above, the concept of transparency is already discussed by some researchers [45,49,52,57], however the typologies do not consider quantitative and accurate measurement of the “visible” space from the external side of the façade.

Hence, more considerations have to be made in measuring and evaluating the degree of visibility of internal spaces adjacent to the façade. Furthermore, in all studies the width of the sidewalk is mostly considered as a part of the evaluation of height to width ratio, or as a part of the setback of the building, without considering it as a possible attribute influencing human behaviour.

Based on the review of the literature, this study proposes a newly defined set of GFFs to be correlated with POSFTFID. This paper seeks to find answers to the following questions: (1) Are spatial GFFs correlated to POSFTFID? (2) If yes, which spatial GFF and which land use are correlated to POSFTFID? (3) To what extent? (4) Which GFFs are strongly correlated with POSFTFID, and which ones with individual users of the POS that may potentially engage in face-to-face interactions?

To answer these questions, this paper analyses data collected in the Nantou Old Town in Shenzhen. This empirical research aims to advance scientific knowledge concerning the analysis of BE spatial features in correlation with POSFTFID, with the proposal of new parameters to be considered for analysis. Moreover, this study seeks to validate three hypotheses concerning this correlation.

**Hypothesis** **1.** *The POS area adjacent to each building is negatively correlated with POSFTFID.*

**Hypothesis** **2.** *The degree of visibility (ratio of indoor visible space to total internal space) of the ground floor is positively correlated to POSFTFID*.

**Hypothesis** **3.** *The degree of exposure (the ratio of POS area to the ground floor area of each building) is negatively correlated to POSFTFID*.

## 3. Materials and Methods

### 3.1. Case Study

This study adopted a case study approach, which is justified by its efficiency in gaining empirical evidence from built environments with high degree of complexity. The ground for choosing a ViC—also known as *chengzhongcun* or “urban village”—in a Chinese fast-growing urban area is constituted by the following considerations: In the last decades China is experiencing an internal migration from rural to urban areas, which is of unprecedented scale worldwide; a great deal of migrants are moving from the countryside to informal areas within the urban territories, such as in ViCs; this population is often economically disadvantaged and with fewer opportunities to access healthcare; in terms of design and variety of spatial forms, ViCs are architectonically-rich spaces, this is particularly true for old villages with stratified layers of construction and modification of the BE; most of the buildings are low-rise with a consequent concentration of public life at the street level; and, the density of users in the OPS of ViCs is generally higher than in other urban areas.

The case study is located in one of the fastest-growing cities of the Greater Bay Area, namely the Special Economic Zone (SEZ) of Shenzhen. In 2014 the Greater Bay Area overpassed Tokyo in terms of population and territorial expansion, becoming the largest mega-region in the world. This region witnessed the earliest ViC in China [58]. In thirty years of evolution, the population of Shenzhen increased from a few hundred thousand in the early 1980s to more than 12 million today. The establishment of a SEZ at the end of the 1970s, concurrently to the Chinese Economic Reform, significantly contributed to attracting foreign investments, transforming Shenzhen into a global manufacturing hub [58].

**Figure 1:**
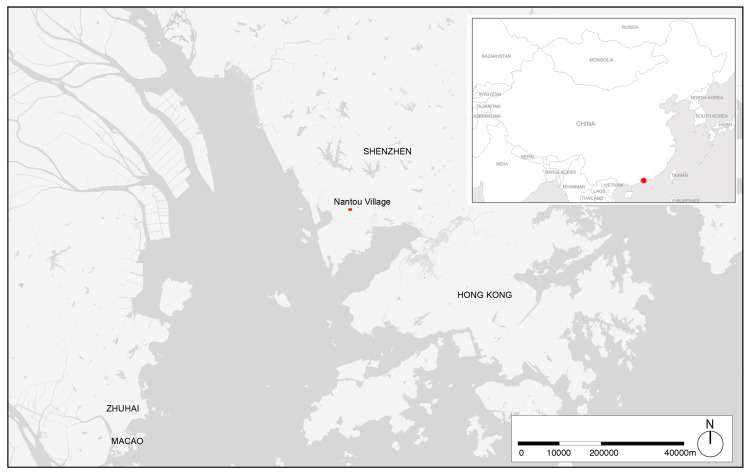
Location of Nantou Old Town within the Greater Bay Area.

With its 1700 years of evolution and a population of around 20,000 inhabitants, Nantou Village (Figure 1) is located in the Nanshan district. It is one of the most ancient ViCs in Shenzhen, still preserving the traditional morphology of the early walled settlements of the region. Nevertheless, the village also underwent several modifications, and from 15 December 2017 to 17 March 2018 it hosted the main venue of the Bi-City Biennale of Urbanism\Architecture (UABB), which contributed to extensive renovation of its BE [59]. Nantou shares with all the other ViCs a high population density, narrow streets mostly serving pedestrians, and the typical configuration of ViCs GFFs. Being encompassed by fast-growing modern urban areas, ViCs are pockets of underdevelopment within the city fabric [60]. They are usually crossed by two orthogonal axes where most of the services are concentrated, while the secondary circulation is embodied by alleys. Usually ViCs have limited access to car circulation, favouring then pedestrian mobility and enhancing the role of the main streets as public spaces. This is also due to the accessibility provided by an extensive mass-transit system in conjunction with informal transportation services [61]. ViCs plots are generally small, which is due to their morphogenesis of upgraded rural ensembles [62].

### 3.2. Analytical Framework

**Figure 2:**
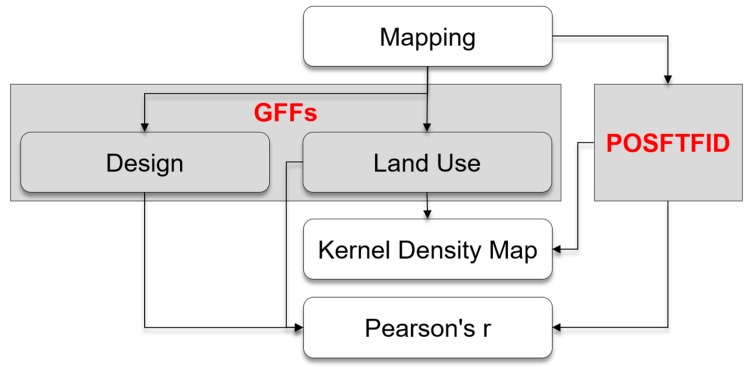
Analytical framework.

The primary aim of this paper is to propose an attempt of methodological advancement in the study of the relationship between face-to-face interaction and the BE, rather than simply providing empirical evidence from a case study. Hence, the case study and the limited dataset are to be understood as testing grounds to prove the feasibility of this approach and to evaluate the limitation for its reproduction in larger spatial and temporal scales. Coherently to the experimental approach herein adopted, popular tools and techniques within the framework of space syntax or other established analytical models of spatial analysis were not considered. Based on the review of the literature, the study is structured in three levels of analysis—mapping spatial settings, mapping behaviours (behavioural mapping), and statistical analysis of the correlation between spatial settings and behaviours (Figure 2). Following, the analysis is performed on two sets of spatial attributes, namely land uses and GFFs design. The first level of analysis is necessary for gathering information on the space itself, the second allows the visualization of the patterns of POSFTFID, and the third aims to provide solid results on a statistical ground.

### 3.3. Observation, Data Collection, and Preparation

Firstly, the researchers conducted extensive on-site systematic observations of main street spaces and activities. The observations intended to provide a deeper understanding of the types and temporal and spatial pattern of use of the POS. To proceed with data collection, local assistants (Masters’ students) were trained. Data were gained in a dual framework. Firstly, GFFs were harvested from available cartographic information and supplemented with on-site mapping and verification. This method has been chosen due to the limitations in the available sources, the method allowing the acquisition of specific spatial attributes at the building scale. The mapped attributes comprised: Internal degree of visibility (m^2^), presence of stairs, size of the adjacent POS (m^2^), and land use. A total of 292 buildings and their adjacent POSs were considered for analysis; these are the buildings with a façade overlooking one of the three main streets of the ViC. Secondly, to perform behaviour mapping, face-to-face interactions in the POS adjacent to each building (georeferenced as a buffer space extending from the outer limit of the building envelop to the centreline of the street) had to be video recorded in order to allow more data accuracy. Subsequently the organization of the dataset was performed. Video recording took place in three different time frames (11:30 am; 2:30–5:30 pm) for two weekend days: Saturday, 6 April 2019 and Sunday, 7 April 2019. Both days presented favourable climatic conditions, the weather being sunny and the average temperature 25 Celsius. The rationale for the choice of two weekend days was the higher pattern of street activities during this period. Observations consisted of persons interacting or potential interactors (individuals not yet engaging in face-to-face interactions) standing or sitting in the POS. Six-hundred and twenty-one observations were collected during the first day, of which 342 were interactors and 279 potential interactors. Five-hundred and seventy-nine observations were collected on the second day, of which 260 were interactors and 319 potential interactors. The total sample size for the whole period is made of 1200 observations, equal to 602 interactors plus 598 potential interactors. Following, every observed individual was captured as a georeferenced point in GIS, and catalogued as interactor (belonging to a group) and potential interactor (standing or sitting alone). Points were categorized as follows: Person sitting, person standing, person in a group, and person alone. The distinction between groups and individuals was based on the literature, specifically stationing individuals were considered as potential interactors. The georeferencing of both behaviours and spatial features allowed the creation of a dataset for further analysis.

### 3.4. Data Analysis

**Figure 3:**
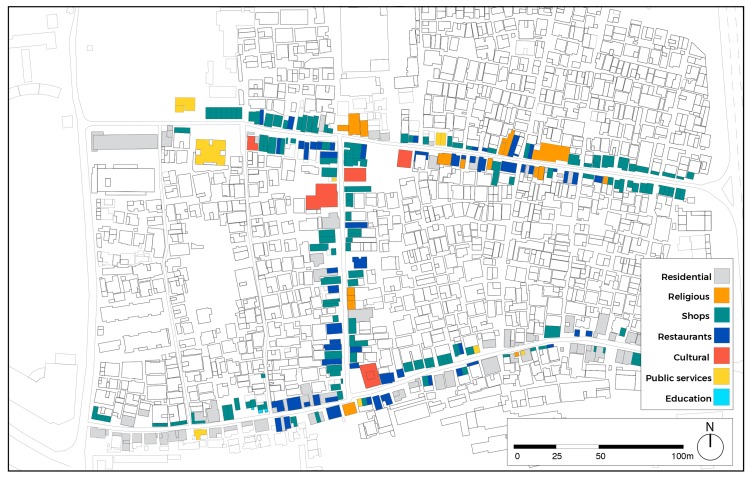
Land use.

**Figure 4:**
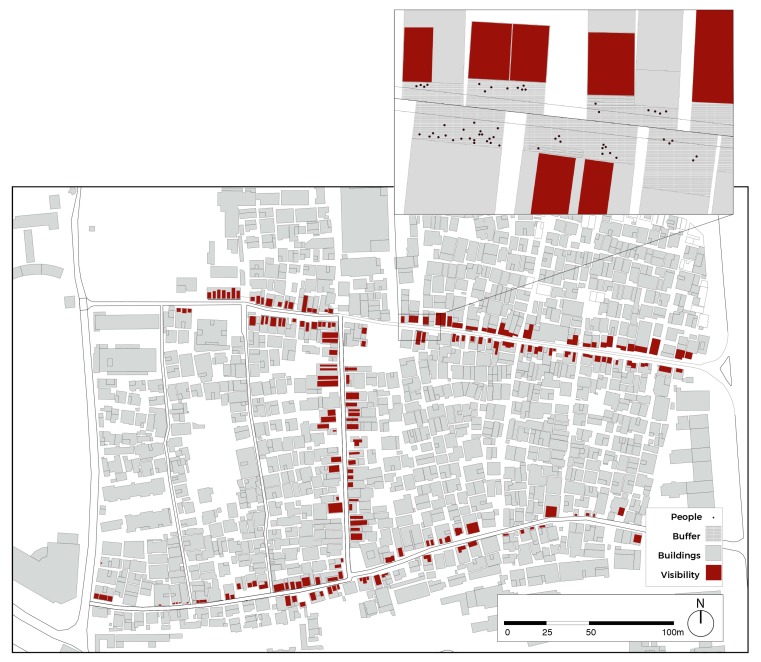
Degree of visibility (m^2^) of GFFs. Top right: Zoom on spatial features, georeferenced points, and Public Open Space (POS) buffers.

Before proceeding with behavioural mapping and statistical analysis, spatial analysis has been carried out to detect land use (Figure 3) and design-related GFFs (Figure 4). Following, behavioural mapping was employed, and the Kernel density tool used to visualize findings. This function of GIS allows the immediate visualization of the patterns of POSFTFID, with the possibility of breaking down POSFTFID by category and time. Behavioural mapping is one of the most effective and feasible methods to understand the relationship between people and place. It is utilized in many fields of research, including environmental science, social science, and educational research. In urban studies, this method stands out for the focus on spatial patterns of activities, allowing investigation in the relationship between physical space and human behaviour. Methods usually adopted to collect data related to human behaviour rely on video recording. In this research, data are collected by adopting a visual snapshot approach. This method consists of recording people standing, sitting, or moving in the selected urban space. The solidity of the results is achieved by video recording in predefined time-frames [63]. In the case of this study, behavioural mapping allowed to draw preliminary considerations in the associations between spatial morphology and human interactions for an initial understanding of urban dynamics at the ground level of the ViC.

The second technique applied consists of the analysis of the datasets prepared earlier in order to statistically verify the effective correlation between the categorized variables. Statistics represent an unavoidable and precious instrument to better understand reality and urbanization dynamics, spatial influence on people’s behaviour, and other essential characteristics of existing urban conditions. The branch of statistics that better fits in this context is correlational research. Specifically, correlational research can describe “the magnitude of the relationship between two variables” [64]. Correlational research is a type of research that involves the measurement of two or more variables. The difference from experimental research consists of the non-intervention of the researcher in the form of a “treatment” [64]. Today, a vast body of literature had already applied an enormous variety of applications of statistical models in urban studies [65]. Within the scope of this paper, Pearson correlation will be performed in order to verify and validate the behaviour mapping results.

Variables considered for Pearson correlation are extrapolated from the GIS dataset previously created and include all spatial features previously detected: Size of ground floor internal space in every building categorized per land use, degree of internal visibility, presence of stairs, and size of adjacent POS; in addition, the ratio between the size of the building and adjacent POS, and ratio of the degree of internal visibility and size of the internal space were also calculated. Furthermore, the datasets include all georeferenced points regarding human interactions, and finally FTFID related to adjacent POS and land use. The formula employed, is the standard sample Pearson correlation coefficient.

## 4. Results and Discussion

### 4.1. Kernel Density Map

**Figure 5:**
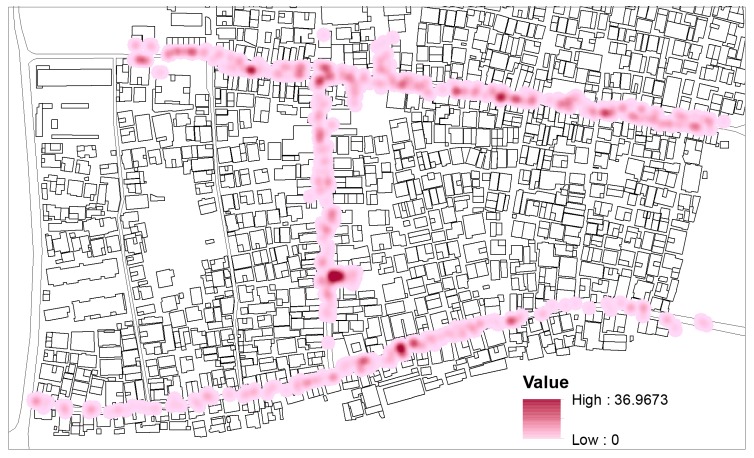
Total Kernel density map showing public open space face-to-face interaction density (POSFTFID).

Firstly, Kernel density maps were generated from GIS in order to visualize the POSFTFID in three different timeframes during the two days of data collection. The maps show a constantly high POSFTFID in specific locations of the POS in all the timeframes. A map comprising all the observed interactions was generated (Figure 5) in order to gain visual evidence of the spatial correlation between POSFTFID and specific land uses. The map shows a higher POSFTFID in correspondence with religious, cultural, and some commercial buildings. Concurrently FTFID in POS adjacent to public services, schools, and residential buildings appears to be relatively low. As an unexpected result, one of the densest points is adjacent to a residential building. This result required further analysis to be verified, consequently statistical analysis was performed considering the total distribution of land use and POSFTFID.

**Figure 6:**
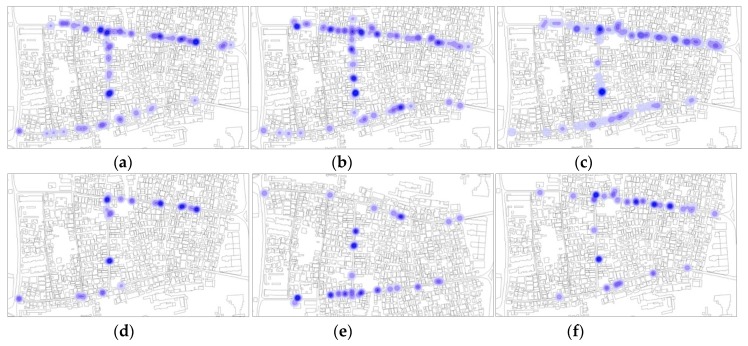
POSFTFID, day one: (**a**) People sitting and standing, 11:30 am; (**b**) people sitting and standing, 2:30 pm; (**c**) people sitting and standing, 5:30 pm; (**d**) people sitting and standing in groups, 11:30 am; (**e**) people sitting and standing in groups, 2:30 pm; (**f**) people sitting and standing in groups, 5:30 pm.

**Figure 7:**
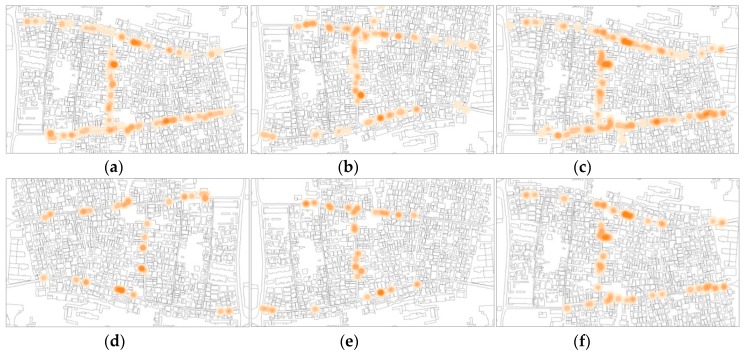
POSFTFID, day two. (**a**) People sitting and standing, 11:30 am; (**b**) people sitting and standing, 2:30 pm; (**c**) people sitting and standing, 5:30 pm; (**d**) people sitting and standing in groups, 11:30 am; (**e**) people sitting and standing in groups, 2:30 pm; (**f**) people sitting and standing in groups, 5:30 pm.

Secondly, following the first map showing the overall merged data, some distinction had to be made to visualize the percentage of individual users of space (non-verbally communicating) compared to persons in groups (already engaged in social interactions). Single maps have been generated distinguishing sitting, standing, individuals, groups, and total number of space users (Figure 6 and Figure 7). The comparison between the maps showed a balance in the percentage between total number of users and individuals. In every time frame and every category, individuals interacting represent a relevant amount of the total number of face-to-face interactions. From the subcategorized maps, a weak sign of ground-level buildings’ functions more correlated to individuals can be observed: Religious buildings and cultural buildings present more POSFTFID. Noticeable, the clustering of POSFTFID in a particular location in front of a residential building appear consistent among several observation periods.

To summarize, the overall results of behavioural mapping refer mostly to function and highlight religious and cultural buildings as potential attractors of people willing to interact with each other while making use of the space. Further analysis has to be performed to verify the residential use and to further analyse the visualized relationships, considering the distribution of land use and spatial attributes of the GFFs in the ViC.

### 4.2. Correlation

#### 4.2.1. Land Use

Behavioural mapping showed some potential relationship between land use-related GFFs and POSFTFID. Further analysis has to be conducted to systematically evaluate the correlation. The dataset allowed the performance of an in-depth statistical analysis. Land use was captured as a bivariate independent variable, subsequently a point-biserial correlation with multiple dichotomous variables was employed. Statistically significant correlations were found only in the case of residential and restaurant/food spaces width, on the total number of observations r equal to –0.221 and 0.150, respectively (Table 2). The breakdown of potential interactors and interactors shows higher values of r in case of potential interactors in both residential and restaurant/food buildings. Noticeably, except by the residential building, results are not statistically significant in the case of interactors. The positive coefficient for POSFTFID and restaurant/food suggests the valuable social role of this type of shopfronts.

#### 4.2.2. Design-Related GFFs

**Figure 8:**
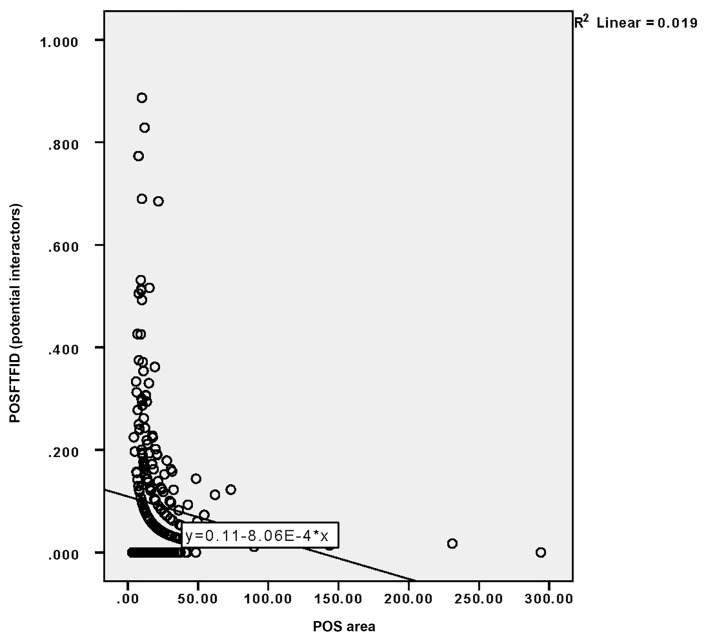
Correlation graph of POSFTFID (potential interactors) (y) and POS area (x).

The correlation between design-related GFFs and POSFTFID cannot be accurately rendered with behavioural mapping. Stairs were recorded as a dichotomous variable in the analytical model, which included continues variables (Table 3). Firstly, POSFTFID has been calculated as a ratio of the included observations (points) on total m^2^ of POS adjacent area. Secondly, the correlation between the POSFTFID and the following design-related GFFs was tested: Ground floor area, indoor space visible from POS, the presence of stairs, and the area of the adjacent POS. In relation to the total number of observations results show statistically significant correlation with stairs (r = 0.182), adjacent POS (r = –0.143), and the degree of visibility (r = 0.145). In the case of stairs, the correlation is relatively strong for potential interactors (r = 0.220). Remarkably, in the case of interactors, the correlation is very weak in all cases, as well as not statistically significant. Noticeably, the correlation between POSFTFID and the area of POS shows a curvilinear development, especially in the case of potential interactors (Figure 8).

### 4.3. Hypotheses Validation

#### 4.3.1. Hypothesis 1

The area of the POS (buffer) adjacent to each building ground floor is negatively correlated with POSFTFID among all categories of POSFTFID (r = −0.140 for potential interactors, r = −0.082 for interactors, r = −0.143 for total number of observations). Despite correlation being weak, the negative Pearson’s r validates the hypothesis. A negative correlation implies that smaller POSs adjacent to buildings, and consequently shorter building façades, are likely to record higher POSFTFID than larger ones. In other terms, the fragmentation of the street façade is positively correlated with POSFTFID.

#### 4.3.2. Hypothesis 2

In order to verify the second hypotheses, the degree of visibility was calculated as the ratio of indoor visible area (m^2^) to the total ground floor area (m^2^). Subsequently, the degree of visibility for each building lined up on the main streets was correlated with POSFTFID. Results showed weak positive Pearson’s r among all categories of POSFTFID (r = 0.131 for potential interactors and r = 0.109 for interactors, and r = 0.145 for total number of observations). The degree of visibility (ratio of indoor visible area to total internal area) of the ground floor is positively correlated to POSFTFID.

#### 4.3.3. Hypothesis 3

With the aim of verifying hypothesis 3, another ratio was calculated, the ratio of POS area to the ground floor area of each building. Subsequently, this ratio, hereafter “degree of exposure”, was correlated with POSFTFID. In this case, Pearson’s r calculated on the total number of observations results negative (r = −0.076). In case of interactors, results show no correlation (r = 0.009). In the case of potential interactionists, the correlation is shown negative (r = −0.150). Although the correlation is being weak, there would be evidence of the degree of exposure being negatively correlated with POSFTFID, or in other terms, that increasing the ratio of outdoor to indoor space would decrease the density of individuals which are likely to engage in face-to-face interactions. However, the results are not statistically significant.

## 5. Conclusions

This study is to be understood as an attempt to offer new methodological advancements in researching the association between GFFs and POSFTFID. Within this aim, the case study of Nantou Village is used as a testing ground in measuring the association. The innovation brought by this paper is related to the inclusion of a new set of variables for the definition of design-related GFFs. Hence, the novelty pertains to the analytical model rather than to the methods employed. Particularly, new concepts such as degree of visibility and the degree of exposure are introduced. Results show an association between POSFTFID and: (1) The area of the POS adjacent to each building, (2) the degree of visibility (ratio of indoor visible space to total internal space) of the ground floor, (3) the presence of stairs. Despite the empirical findings demonstrating a weak correlation, it is safe to advance that the fragmentation of the street front, more visible indoor spaces, and buildings with stairs are likely to be associated with a higher density of human face-to-face interaction. In addition, the correlation of land use and POSFTFID, present more mixed results, however it indicates a positive association between restaurant or food shops and POSFTFID, highlighting the social importance of food.

Nevertheless, this study presents a series of limitations that should be addressed by future works. Firstly, the sample size is relatively small for behavioural analysis (*N* = 1200). A larger sample size will enable higher data accuracy and further investigation with the application of more advanced statistical techniques. Moreover, a larger sample may also address bias, specifically data must be collected in a longer period, with a balanced presence of working and non-working days. Considering different case studies, not only from China, but also from some other regions of the world, will enable more robust findings, as well as the possibility of comparative studies. In regard to the hereby proposed degree of enclosure, future studies may also consider the third dimension (height), rather than simply the ground floor area. Eventually, the third dimension should be considered also in relation to more complex building environments which develop in height, such as high-density urban environments in which grade-separation is applied.

Despite the limitations, this study is an important addition to the body of knowledge concerned with the relationship between BE and public health. Both analytical framework and models here adopted are proposing novel approaches in the study of this relationship. Furthermore, the findings presented in this study are extremely relevant for enabling architects, urban designers, planners, and policymakers to produce pedestrian streets (building ground floors and adjacent POS) that can be effective in addressing issues of public health related to face-to-face interaction. Additionally, the chosen case study is bringing new public attention to the valuable role of Chinese ViCs in providing ideal settings for the interaction of disadvantaged social groups. Noticeably, the focus on the GFFs hereby proposed aims to direct scholarly and public attention on the significance of these spatial settings, as suggested also by recent studies [66].

## Figures and Tables

**Table 1:** Literature investigating the association between spatial features of ground floor features (GFFs) and human behaviour or perceptions of space.

Type	Features	Description	Observable Variables	Measurement	Findings	Source
Architectural articulation	Façade articulation	-	Texture, size, colour, façade components, and shape	Building shape (no rectangular silhouettes), pedestrian/car setbacks	Creation of a more human scale environment, creation of stationary activities place through subspace in the façade	[33,44,45]
	Rhythm	-	Vertical and horizontal lines in the façade	Height (building) to width (sidewalk) ratio, proportion of first floor with windows, number of ground-level doors facing the street, average sidewalk width	Attraction of pedestrians’ attention to ground-level, impact on walking speed (slower)	[45,47]
	Scale	-	Presence of narrow units in the architectural façade		Increase in façade niches and contribution to pedestrian activities	
Permeability	Physical permeability	Physical access to a space		Degree of street-front permeability on the block-segment, number of ground-level entrances, proportion of active uses	Attraction of pedestrians’ attention to explore the ground-level façade Increase in pedestrian activities in front of higher transparency façades	[48]
	Transparency	Degree to which people can see human activity or what lies beyond the edge from the street	Degree of transparency (impermeable/opaque /transparent)	Proportion first floor with windows		[32,33,38,45]
Personalization		Degree to which a street appears to be modified and claimed by residents or store owners	Presence of decorative elements and signs, addition of seating and shading, landscaped areas, Dominant building colours	-	Promotion of pedestrian activities at the ground-level, contribution to sense of security to users	[44,48,50]
Enclosure		Degree to which public space is visually defined by buildings, walls, trees		Height to width ratio, number of street furniture and vegetation, proportion sky ahead	People preference for enclosed spaces	[25,45,48,52]
Land use		Diversity in the ground-level land use	Presence of commercial local stores,	number of independent businesses on the block-segment, number of outdoor dining, entropy index	Promotion of pedestrian activities on the streets, contribution to sense of security to users (eyes on the street)	[30,48,53,54]
Amenities provision		Number of seating space and shaded areas	Seating space and shaded areas	number of public (non-commercial) or commercial seating on the block-segment, percentage shade and shelter from trees and canopies on the block-segment	Promotion of pedestrian activities on the streets, contribution to pedestrians’ comfort Increase in sense of human scale	[31,32,55]

**Table 2:** Pearson’s r (point-biserial correlation) of land use and POSFTFID.

Land Use	Potential Interactor	Sig. (2-Tailed)	Interactor	Sig. (2-Tailed)	Total	Sig. (2-Tailed)	*N*
Residential	−0.227 **	0.000	−0.124 *	0.034	−0.221 **	0.000	292
Religious	−0.030	0.607	0.071	0.223	0.028	0.631	292
Retail	0.099	0.091	0.067	0.255	0.117 *	0.046	292
Restaurant/Food	0.185 **	0.001	0.076	0.194	0.150 *	0.010	292
Cultural	−0.016	0.788	0.032	0.586	0.012	0.835	292
Public Service	0.059	0.317	−0.066	0.257	−0.080	0.171	292
Educational	−0.053	0.364	−0.013	0.828	−0.040	0.493	292

**. Correlation is significant at the 0.01 level (2-tailed). *. Correlation is significant at the 0.05 level (2-tailed).

**Table 3:** Pearson’s r related to design-related GFFs and POSFTFID.

Design	Unit	Potential Interactor	Sig. (2-Tailed)	Interactor	Sig. (2-Tailed)	Total	Sig. (2-Tailed)	*N*
Internal space	m^2^	−0.088	0.135	−0.067	0.252	−0.105	0.073	292
Visible space	m^2^	0.059	0.318	0.053	0.362	0.064	0.274	292
Stairs	dummy	0.220 **	0.000	0.089	0.129	0.182 **	0.002	292
POS (buffer)	m^2^	−0.140 *	0.017	−0.82	0.161	−0.143 *	0.015	292
Visible/Inter. space	ratio	0.131 *	0.025	0.109	0.064	0.145 *	0.013	292
POS/Inter. space	ratio	−0.150	0.010	0.009	0.882	−0.076	0.198	292

**. Correlation is significant at the 0.01 level (2-tailed). *. Correlation is significant at the 0.05 level (2-tailed).
